# *catena*-Poly[[bis­(2-hydr­oxy-2-phenyl­acetato-κ^2^ *O* ^1^,*O* ^2^)zinc(II)]-μ-1,2-di-4-pyridylethane-κ^2^ *N*:*N*′]

**DOI:** 10.1107/S1600536809030281

**Published:** 2009-08-08

**Authors:** Seung Man Yu, Dong Hoon Shin, Pan-Gi Kim, Cheal Kim, Youngmee Kim

**Affiliations:** a Department of Fine Chemistry, and Eco-Products and Materials Education Center, Seoul National University of Technology, Seoul 139-743, Republic of Korea; b Department of Forest & Environmental Resources, Kyungpook National University, Sangju 742-711, Republic of Korea; c Department of Chemistry and Nano Science, Ewha Womans University, Seoul 120-750, Republic of Korea

## Abstract

The title compound, [Zn(C_8_H_6_O_3_)_2_(C_12_H_12_N_2_)]_*n*_, consists of [Zn(Hopa)_2_] (H_2_opa = 2-hydr­oxy-2-phenyl­acetic acid or mandelic acid) units bridged by 1,2-di-4-pyridylethane (bpe) ligands, forming a polymeric chain developing parallel to the *b* axis. The bridging bpe ligand is arranged around a twofold axis passing through the middle of the ethane C—C bond. The geometry around the Zn^II^ ion is distorted octa­hedral, constructed by four O atoms from two Hopa^−^ ligands and two N atoms from two bridging bpe ligands. O—H⋯O hydrogen bonds link the chains, forming a three-dimensional network.

## Related literature

Transition metal ions are the major cationic contributors to the inorganic composition of natural water and biological fluids, see: Daniele *et al.* (2008 ▶). For related structures, see: Balboa *et al.* (2008 ▶); Beghidja *et al.* (2005 ▶); Hao *et al.* (2009 ▶); Lee *et al.* (2008 ▶); Park *et al.* (2008 ▶); Shin *et al.* (2009 ▶); Wermester *et al.* (2007 ▶); Yu *et al.* (2008 ▶). 
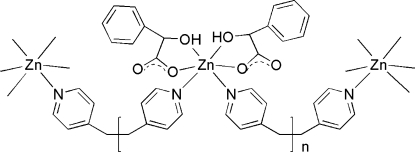


## Experimental

### 

#### Crystal data

[Zn(C_8_H_6_O_3_)_2_(C_12_H_12_N_2_)]
*M* *_r_* = 551.90
Hexagonal, 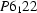

*a* = 11.1360 (6) Å
*c* = 33.110 (3) Å
*V* = 3555.9 (4) Å^3^
*Z* = 6
Mo *K*α radiation
μ = 1.09 mm^−1^
*T* = 293 K
0.10 × 0.05 × 0.05 mm

#### Data collection

Bruker SMART CCD diffractometer
Absorption correction: multi-scan (*SADABS*; Bruker, 1997 ▶) *T*_min_ = 0.933, *T*_max_ = 0.944
17715 measured reflections
2347 independent reflections
2045 reflections with *I* > 2σ(*I*)
*R*_int_ = 0.077

#### Refinement

*R*[*F* ^2^ > 2σ(*F* ^2^)] = 0.032
*wR*(*F* ^2^) = 0.068
*S* = 1.04
2347 reflections
168 parameters
1 restraint
H-atom parameters constrained
Δρ_max_ = 0.22 e Å^−3^
Δρ_min_ = −0.21 e Å^−3^
Absolute structure: Flack (1983 ▶), 870 Friedel pairs
Flack parameter: −0.002 (16)

### 

Data collection: *SMART* (Bruker, 1997 ▶); cell refinement: *SAINT* (Bruker, 1997 ▶); data reduction: *SAINT*; program(s) used to solve structure: *SHELXS97* (Sheldrick, 2008 ▶); program(s) used to refine structure: *SHELXL97* (Sheldrick, 2008 ▶); molecular graphics: *PLATON* (Spek, 2009 ▶); software used to prepare material for publication: *SHELXL97*.

## Supplementary Material

Crystal structure: contains datablocks I, global. DOI: 10.1107/S1600536809030281/dn2478sup1.cif

Structure factors: contains datablocks I. DOI: 10.1107/S1600536809030281/dn2478Isup2.hkl

Additional supplementary materials:  crystallographic information; 3D view; checkCIF report

## Figures and Tables

**Table 1:** Hydrogen-bond geometry (Å, °)

*D*—H⋯*A*	*D*—H	H⋯*A*	*D*⋯*A*	*D*—H⋯*A*
O13—H13*O*⋯O12^i^	0.85	1.77	2.619 (3)	173

Symmetry code: (i) 
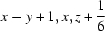
.

**Fig. 1.**
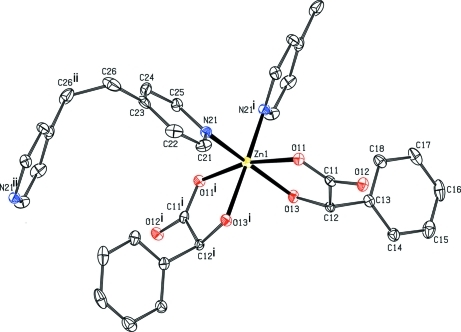
View of compound I with the atom labeling scheme. Displacement ellipsoids are drawn at the 30° probability level. H atoms have been omitted for clarity. [Symmetry codes: (i) x, x-y+1, -z+1/6; (ii) x, x-y+2, -z+1/6].

## supplementary crystallographic information

### Comment

A great attention has been paid to transition metal ions as the major cation contributors to the biologically active molecules such as amino acids, proteins, sugars, nucleotides *etc* (Daniele, *et al.*, 2008). This interest has driven us to study on the interaction of the transition metal ions with fulvic acids or humic acids. As models to examine the interaction, therefore, we have previously used copper(II) and zinc(II) benzoates as building blocks and reported the structures of copper(II) and zinc(II) benzoates with quinoxaline, 6-methylquinoline, 3-methylquinoline, and di-2-pyridyl ketone(Lee *et al.*, 2008; Yu *et al.*, 2008; Park *et al.*, 2008; Shin *et al.*, 2009).

Mandelic acid (2-hydroxy-2-phenylacetic acid, H_2_opa) is also one of the simplest bioactive molecules exhibiting a variety of coordinating and supramolecular interaction abilities (Balboa *et al.*, 2008; Beghidja *et al.*,2005; Hao *et al.*, 2009; Wermester *et al.*, 2007). In order to study the interaction of the biologically active molecule mandelic acid with zinc(II) ion, in the present work, we have employed zinc(II) mandelate as a building block and 1,2-di-4-pyridylethane (bpe) as a ligand. We report herein the structure of new zinc(II) mandelate with 1,2-di-4-pyridylethane.

The crystal structure contains [Zn(Hopa)_2_] units bridged by bpe ligands forming a polymeric chain developping parallel to the b axis. The bridging 1,2-di-4-pyridylethane (bpe) ligand is arranged around a two fold axis going through the middle of C26—C26^ii^ bond (symmetry code: (ii) x, x-y+2, -z+1/6). The geometry around the Zn^II^ ion is distorted octahedral constructed by four oxygen atoms from two Hopa^-^ and two nitrogen atoms from two bridging bpe ligands (Fig. 1). The occurence of O-H···O hydrogen bonds links the chains to form a three dimensionnal network.

### Experimental

38.0 mg (0.125 mmol) of Zn(NO_3_)_2_^.^6H_2_O and 38.4 mg (0.25 mmol) of 2-hydroxy-2-phenylacetic acid were dissolved in 4 ml water and carefully layered by 4 ml solution of a mixture of acetone, methanol and ethanol (2/2/2) of 1,2-di-4-pyridylethane ligand (46.0 mg, 0.25 mmol). Suitable crystals of the title compoundfor X-ray analysis were obtained in a few weeks.

### Refinement

All H atoms attached to C atoms were fixed geometrically and treated as riding with C—H = 0.98 Å (methyne),0.97 Å (methylene) or 0.93 Å (aromatic) with U_iso_(H) = 1.2U_eq_(C). H atom attached to O was located in difference Fourier maps and included in the subsequent refinement using restraints (O-H= 0.85 (1)Å) with U_iso_(H) = 1.5U_eq_(O). In the last stage of refinement, it was treated as riding on its parent atom.

### Crystal data

**Table:**

[Zn(C_8_H_6_O_3_)_2_(C_12_H_12_N_2_)]	*D*_x_ = 1.546 Mg m^−^^3^
*M**_r_* = 551.90	Mo *K*α radiation, λ = 0.71073 Å
Hexagonal, *P*6_1_22	Cell parameters from 1751 reflections
Hall symbol: P 61 2 (0 0 -1)	θ = 2.2–18.9°
*a* = 11.1360 (6) Å	µ = 1.09 mm^−^^1^
*c* = 33.110 (3) Å	*T* = 293 K
*V* = 3555.9 (4) Å^3^	Rod, colorless
*Z* = 6	0.10 × 0.05 × 0.05 mm
*F*(000) = 1716	

### Data collection

**Table:**

Bruker SMART CCD diffractometer	2347 independent reflections
Radiation source: fine-focus sealed tube	2045 reflections with *I* > 2σ(*I*)
graphite	*R*_int_ = 0.077
φ and ω scans	θ_max_ = 26.0°, θ_min_ = 2.1°
Absorption correction: multi-scan (*SADABS*; Bruker, 1997)	*h* = −13→13
*T*_min_ = 0.933, *T*_max_ = 0.944	*k* = −11→13
17715 measured reflections	*l* = −40→28

### Refinement

**Table:**

Refinement on *F*^2^	Secondary atom site location: difference Fourier map
Least-squares matrix: full	Hydrogen site location: inferred from neighbouring sites
*R*[*F*^2^ > 2σ(*F*^2^)] = 0.032	H-atom parameters constrained
*wR*(*F*^2^) = 0.068	*w* = 1/[σ^2^(*F*_o_^2^) + (0.016*P*)^2^ + 1.0575*P*] where *P* = (*F*_o_^2^ + 2*F*_c_^2^)/3
*S* = 1.04	(Δ/σ)_max_ < 0.001
2347 reflections	Δρ_max_ = 0.22 e Å^−^^3^
168 parameters	Δρ_min_ = −0.21 e Å^−^^3^
1 restraint	Absolute structure: Flack (1983), 870 Friedel pairs
Primary atom site location: structure-invariant direct methods	Flack parameter: −0.002 (16)

### Special details

**Table:**

Geometry. All esds (except the esd in the dihedral angle between two l.s. planes) are estimated using the full covariance matrix. The cell esds are taken into account individually in the estimation of esds in distances, angles and torsion angles; correlations between esds in cell parameters are only used when they are defined by crystal symmetry. An approximate (isotropic) treatment of cell esds is used for estimating esds involving l.s. planes.
Refinement. Refinement of *F*^2^ against ALL reflections. The weighted *R*-factor *wR* and goodness of fit *S* are based on *F*^2^, conventional *R*-factors *R* are based on *F*, with *F* set to zero for negative *F*^2^. The threshold expression of *F*^2^ > σ(*F*^2^) is used only for calculating *R*-factors(gt) *etc*. and is not relevant to the choice of reflections for refinement. *R*-factors based on *F*^2^ are statistically about twice as large as those based on *F*, and *R*- factors based on ALL data will be even larger.

### Fractional atomic coordinates and isotropic or equivalent isotropic displacement parameters (Å^2^)

**Table:**

	*x*	*y*	*z*	*U*_iso_*/*U*_eq_	
Zn1	0.73603 (4)	0.86802 (2)	0.0833	0.01944 (12)	
O11	0.76202 (18)	0.84579 (18)	0.02239 (5)	0.0216 (4)	
O12	0.89644 (18)	0.82448 (18)	−0.02384 (5)	0.0245 (5)	
O13	0.89365 (17)	0.81243 (17)	0.08319 (5)	0.0233 (4)	
H13O	0.9568	0.8433	0.1013	0.035*	
N21	0.5928 (2)	0.9386 (2)	0.07376 (7)	0.0223 (5)	
C11	0.8619 (3)	0.8302 (2)	0.01183 (8)	0.0191 (6)	
C12	0.9540 (3)	0.8194 (3)	0.04470 (8)	0.0179 (6)	
H12	1.0448	0.9043	0.0438	0.022*	
C13	0.9761 (3)	0.6975 (3)	0.03869 (8)	0.0201 (6)	
C14	1.1078 (3)	0.7159 (3)	0.03588 (9)	0.0284 (7)	
H14	1.1839	0.8052	0.0368	0.034*	
C15	1.1293 (4)	0.6038 (4)	0.03167 (10)	0.0394 (8)	
H15	1.2189	0.6185	0.0295	0.047*	
C16	1.0181 (4)	0.4716 (4)	0.03078 (10)	0.0423 (9)	
H16	1.0316	0.3960	0.0283	0.051*	
C17	0.8862 (4)	0.4520 (3)	0.03359 (9)	0.0382 (8)	
H17	0.8105	0.3624	0.0328	0.046*	
C18	0.8639 (3)	0.5636 (3)	0.03754 (8)	0.0280 (6)	
H18	0.7741	0.5486	0.0394	0.034*	
C21	0.6235 (3)	1.0429 (3)	0.04786 (9)	0.0310 (8)	
H21	0.7018	1.0742	0.0317	0.037*	
C22	0.5445 (3)	1.1057 (3)	0.04412 (10)	0.0357 (8)	
H22	0.5682	1.1757	0.0252	0.043*	
C23	0.4294 (3)	1.0642 (3)	0.06862 (10)	0.0289 (7)	
C24	0.4001 (3)	0.9599 (3)	0.09618 (9)	0.0288 (7)	
H24	0.3251	0.9299	0.1136	0.035*	
C25	0.4832 (3)	0.9009 (3)	0.09751 (8)	0.0252 (6)	
H25	0.4613	0.8304	0.1161	0.030*	
C26	0.3364 (3)	1.1263 (3)	0.06523 (12)	0.0520 (11)	
H26A	0.2422	1.0517	0.0607	0.062*	
H26B	0.3641	1.1857	0.0416	0.062*	

### Atomic displacement parameters (Å^2^)

**Table:**

	*U*^11^	*U*^22^	*U*^33^	*U*^12^	*U*^13^	*U*^23^
Zn1	0.0209 (2)	0.01972 (17)	0.0181 (2)	0.01044 (12)	0.000	−0.00108 (18)
O11	0.0248 (10)	0.0247 (10)	0.0185 (10)	0.0148 (8)	−0.0010 (8)	0.0008 (8)
O12	0.0311 (12)	0.0325 (11)	0.0154 (10)	0.0200 (9)	0.0007 (9)	0.0017 (8)
O13	0.0270 (10)	0.0352 (10)	0.0135 (9)	0.0198 (9)	−0.0050 (9)	−0.0048 (8)
N21	0.0218 (12)	0.0224 (12)	0.0213 (14)	0.0101 (10)	0.0011 (10)	0.0006 (10)
C11	0.0231 (13)	0.0141 (14)	0.0176 (15)	0.0074 (12)	−0.0024 (13)	−0.0004 (11)
C12	0.0190 (13)	0.0189 (13)	0.0142 (14)	0.0083 (10)	−0.0012 (11)	−0.0025 (11)
C13	0.0274 (15)	0.0231 (15)	0.0113 (13)	0.0137 (12)	−0.0034 (11)	0.0004 (12)
C14	0.0297 (17)	0.0309 (16)	0.0296 (18)	0.0189 (14)	−0.0055 (13)	−0.0065 (14)
C15	0.048 (2)	0.057 (2)	0.0319 (19)	0.040 (2)	−0.0055 (17)	−0.0054 (17)
C16	0.080 (3)	0.049 (2)	0.0260 (18)	0.053 (2)	−0.0044 (18)	−0.0044 (16)
C17	0.062 (2)	0.0242 (17)	0.0226 (17)	0.0172 (17)	−0.0001 (16)	−0.0020 (14)
C18	0.0350 (16)	0.0237 (16)	0.0215 (15)	0.0117 (14)	−0.0015 (14)	−0.0003 (13)
C21	0.0241 (16)	0.0359 (18)	0.0286 (18)	0.0118 (14)	0.0005 (13)	0.0089 (14)
C22	0.0329 (18)	0.0310 (18)	0.0393 (19)	0.0131 (16)	−0.0083 (15)	0.0094 (14)
C23	0.0298 (17)	0.0265 (15)	0.0344 (19)	0.0172 (14)	−0.0179 (14)	−0.0153 (14)
C24	0.0275 (17)	0.0359 (18)	0.0293 (17)	0.0206 (14)	0.0010 (13)	−0.0039 (14)
C25	0.0306 (16)	0.0239 (16)	0.0219 (15)	0.0143 (12)	0.0033 (13)	0.0018 (12)
C26	0.039 (2)	0.0319 (17)	0.093 (3)	0.0242 (16)	−0.0356 (19)	−0.0234 (19)

### Geometric parameters (Å, °)

**Table:**

Zn1—O11	2.0707 (17)	C15—C16	1.371 (5)
Zn1—O11^i^	2.0707 (17)	C15—H15	0.9300
Zn1—N21	2.125 (2)	C16—C17	1.376 (5)
Zn1—N21^i^	2.125 (2)	C16—H16	0.9300
Zn1—O13^i^	2.1332 (17)	C17—C18	1.390 (4)
Zn1—O13	2.1332 (17)	C17—H17	0.9300
O11—C11	1.258 (3)	C18—H18	0.9300
O12—C11	1.254 (3)	C21—C22	1.376 (4)
O13—C12	1.425 (3)	C21—H21	0.9300
O13—H13O	0.8543	C22—C23	1.386 (4)
N21—C25	1.332 (3)	C22—H22	0.9300
N21—C21	1.344 (3)	C23—C24	1.381 (4)
C11—C12	1.541 (4)	C23—C26	1.511 (4)
C12—C13	1.509 (4)	C24—C25	1.378 (4)
C12—H12	0.9800	C24—H24	0.9300
C13—C14	1.379 (4)	C25—H25	0.9300
C13—C18	1.387 (4)	C26—C26^ii^	1.519 (7)
C14—C15	1.390 (4)	C26—H26A	0.9700
C14—H14	0.9300	C26—H26B	0.9700
			
O11—Zn1—O11^i^	166.10 (10)	C13—C14—H14	119.3
O11—Zn1—N21	94.27 (8)	C15—C14—H14	119.3
O11^i^—Zn1—N21	94.75 (8)	C16—C15—C14	119.8 (3)
O11—Zn1—N21^i^	94.75 (8)	C16—C15—H15	120.1
O11^i^—Zn1—N21^i^	94.27 (8)	C14—C15—H15	120.1
N21—Zn1—N21^i^	98.92 (11)	C15—C16—C17	119.3 (3)
O11—Zn1—O13^i^	92.80 (7)	C15—C16—H16	120.4
O11^i^—Zn1—O13^i^	77.21 (7)	C17—C16—H16	120.4
N21—Zn1—O13^i^	86.60 (7)	C16—C17—C18	121.2 (3)
N21^i^—Zn1—O13^i^	170.28 (8)	C16—C17—H17	119.4
O11—Zn1—O13	77.21 (7)	C18—C17—H17	119.4
O11^i^—Zn1—O13	92.80 (7)	C13—C18—C17	119.8 (3)
N21—Zn1—O13	170.28 (8)	C13—C18—H18	120.1
N21^i^—Zn1—O13	86.60 (7)	C17—C18—H18	120.1
O13^i^—Zn1—O13	89.11 (9)	N21—C21—C22	123.1 (3)
C11—O11—Zn1	118.26 (16)	N21—C21—H21	118.5
C12—O13—Zn1	114.85 (15)	C22—C21—H21	118.5
C12—O13—H13O	109.5	C21—C22—C23	119.8 (3)
Zn1—O13—H13O	120.8	C21—C22—H22	120.1
C25—N21—C21	116.5 (2)	C23—C22—H22	120.1
C25—N21—Zn1	122.35 (18)	C24—C23—C22	117.2 (3)
C21—N21—Zn1	120.11 (19)	C24—C23—C26	120.4 (3)
O12—C11—O11	125.7 (3)	C22—C23—C26	122.4 (3)
O12—C11—C12	115.4 (2)	C25—C24—C23	119.3 (3)
O11—C11—C12	118.9 (2)	C25—C24—H24	120.4
O13—C12—C13	110.7 (2)	C23—C24—H24	120.4
O13—C12—C11	108.8 (2)	N21—C25—C24	124.0 (3)
C13—C12—C11	113.0 (2)	N21—C25—H25	118.0
O13—C12—H12	108.1	C24—C25—H25	118.0
C13—C12—H12	108.1	C23—C26—C26^ii^	115.7 (3)
C11—C12—H12	108.1	C23—C26—H26A	108.4
C14—C13—C18	118.5 (3)	C26^ii^—C26—H26A	108.4
C14—C13—C12	121.0 (3)	C23—C26—H26B	108.4
C18—C13—C12	120.4 (2)	C26^ii^—C26—H26B	108.4
C13—C14—C15	121.4 (3)	H26A—C26—H26B	107.4

Symmetry codes: (i) *x*, *x*−*y*+1, −*z*+1/6; (ii) *x*, *x*−*y*+2, −*z*+1/6.

### Hydrogen-bond geometry (Å, °)

**Table:**

*D*—H···*A*	*D*—H	H···*A*	*D*···*A*	*D*—H···*A*
O13—H13O···O12^iii^	0.85	1.77	2.619 (3)	173

Symmetry codes: (iii) *x*−*y*+1, *x*, *z*+1/6.
